# Dissecting Electronic-Structural Transitions in the Nitrogenase MoFe Protein P-Cluster during Reduction

**DOI:** 10.1021/jacs.1c13311

**Published:** 2022-03-22

**Authors:** Bryant Chica, Jesse Ruzicka, Lauren M. Pellows, Hayden Kallas, Effie Kisgeropoulos, Gregory E. Vansuch, David W. Mulder, Katherine A. Brown, Drazenka Svedruzic, John W. Peters, Gordana Dukovic, Lance C. Seefeldt, Paul W. King

**Affiliations:** † Biosciences Center, National Renewable Energy Laboratory, Golden, Colorado 80401, United States; ‡ Department of Chemistry, University of Colorado Boulder, Boulder, Colorado 80309, United States; § Department of Chemistry and Biochemistry, Utah State University, Logan, Utah 84322, United States; ∥ Institute of Biological Chemistry, Washington State University, Pullman, Washington 99163, United States; ⊥ Renewable and Sustainable Energy Institute (RASEI), University of Colorado Boulder, Boulder, Colorado 80309, United States; # Materials Science and Engineering, University of Colorado Boulder, Boulder, Colorado 80303, United States

## Abstract

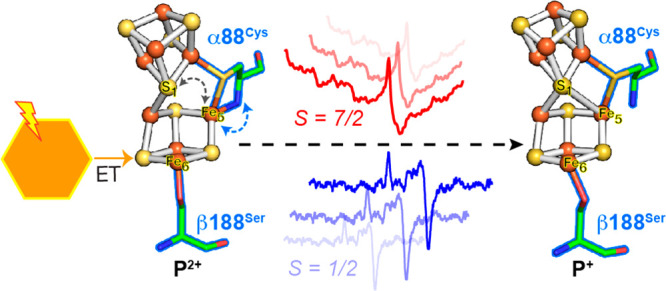

The [8Fe-7S] P-cluster of nitrogenase MoFe protein mediates electron transfer from nitrogenase Fe protein during the catalytic production of ammonia. The P-cluster transitions between three oxidation states, P^N^, P^+^, P^2+^ of which P^N^↔P^+^ is critical to electron exchange in the nitrogenase complex during turnover. To dissect the steps in formation of P^+^ during electron transfer, photochemical reduction of MoFe protein at 231–263 K was used to trap formation of P^+^ intermediates for analysis by EPR. In complexes with CdS nanocrystals, illumination of MoFe protein led to reduction of the P-cluster P^2+^ that was coincident with formation of three distinct EPR signals: *S* = 1/2 axial and rhombic signals, and a high-spin *S* = 7/2 signal. Under dark annealing the axial and high-spin signal intensities declined, which coincided with an increase in the rhombic signal intensity. A fit of the time-dependent changes of the axial and high-spin signals to a reaction model demonstrates they are intermediates in the formation of the P-cluster P^+^ resting state and defines how spin-state transitions are coupled to changes in P-cluster oxidation state in MoFe protein during electron transfer.

Nitrogenase is a two-component enzyme that catalyzes the conversion of dinitrogen to ammonia. Under ideal reaction conditions, the Mo-dependent form of nitrogenase, composed of Fe protein and MoFe protein, catalyzes N_2_ reduction to ammonia according to eq 1:^1^
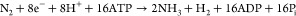
1

During turnover, the electrons required for N_2_ reduction are transferred from Fe protein to MoFe protein, which is an α_2_β_2_ tetramer that coordinates two sets of unique metal clusters. The [8Fe-7S] P-cluster functions in electron transfer with Fe protein, and the [7Fe-9S-1Mo-C-Homocitrate] iron–molybdenum cofactor (FeMo-co) functions as the site of N_2_ reduction.^2^

One of the unique aspects of how nitrogenase catalyzes ammonia production is the electron transfer process.^3,4^ In the catalytic cycle, the P-cluster forms a metastable intermediate oxidation state, P^+^ ([7Fe^II^Fe^III^-7S]^+1^), that is rapidly reduced (*k* > 1700 s^–1^)^3^ during electron transfer. In addition to P^+^, the P-cluster forms two stable oxidation states, P^N^ ([8Fe^II^-7S]^0^ and P^2+^ ([6Fe^II^2Fe^III^-7S]^+2^) (Figure 1).^5^ Transitions between P-cluster states involve extensive structural changes, including a switch in Fe-coordination of the central sulfide (S_1_) that bridges the two [4Fe-3S] subclusters, and amide nitrogen coordination to Fe_5_ by α-88 cysteine (α-88Cys) and β-188 serine (β-188Ser) oxygen coordination to Fe_6_. Recently, the X-ray structure of MoFe protein was solved with the P-cluster poised in the P^+^ state, with an intermediate structural arrangement between P^2+^ and P^N^ (Figure 1).^6^ In the P^+^ state, the S_1_ sulfide is pentacoordinate and β-188Ser coordinates Fe_6_. The observation of structural changes in the MoFe protein P-cluster has been incorporated into conformational gating^7^ and mechanical coupling^8,9^ electron transfer models. The model predicts that motions near the P-cluster and β-188Ser are coupled to “switch regions” in the Fe protein that steer structural interactions within the nitrogenase complex to enable electron delivery.^9^

**Figure 1:**
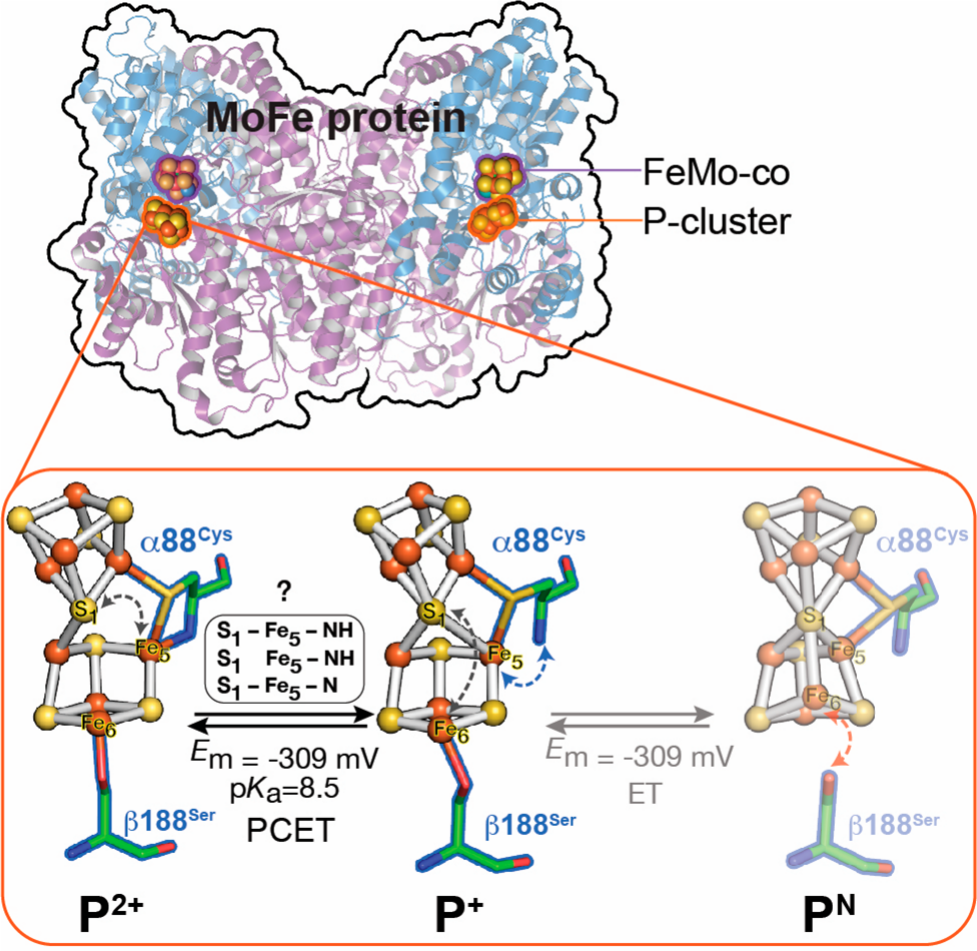
MoFe protein P-cluster oxidation state structures for P^2+^, P^+^, and P^N^. The α-88^Cys^ (P^2+^↔P^+^) and β-188^Ser^ (P^+^↔P^N^) ligands that undergo redox-coupled coordination changes to the P-cluster are shown. The P^2+^↔P^+^ transition involves exchange of the S_1_–Fe_5_ thiolate bond (gray arrow) and α-88^Cys^ amide bond at Fe_5_ (blue arrow) and proceeds via proton-coupled electron transfer (PCET), where changes in bonding (box) may lead to different conformers during electron transfer. The P^+^↔P^N^ transition involves exchange of the S_1_–Fe_6_ thiolate bond and β-188^Ser^ serine hydroxylate bond at Fe_6_ (red arrow). *E*_m_ = −309 mV at pH 8 for both transitions.^11^ PDB Codes: P^2+^, 2MIN; P^+^, 6CDK; P^N^, 3MIN.

The structural rearrangements of the P-cluster in different oxidation states also coincide with changes in spin states and EPR properties. P^2+^ is an integer spin state, likely *S* = 4, and gives rise to an EPR signal at *g* = 11.8,^10,11^ whereas P^N^ is an *S* = 0 spin state and EPR-silent. The P^+^ oxidation state has a rhombic, *S* = 1/2 EPR signal at *g* = 2.05, 1.94, 1.81 that shifts to *g* = 2.03, 1.97, 1.93 when β-188Ser is substituted by Cys.^12,13^ Additional magnetic signals associated with the P^+^ oxidation state include an *S* = 1/2 signal with *g* = 2.00 and 1.89,^13,14^ and low-field *S* = 5/2 signals^12,13^ (Table S1). Variations in P^+^ magnetic states have been observed in MoFe protein under different redox titration conditions (Table S2).^13−15^ Whether these states have a functional role in electron transfer in MoFe protein remains unclear. Recently, the structural and magnetic configurations of the P-cluster oxidation states were shown to coincide with profound differences in the density of low lying electronic states, implying there is a deeper relationship between the electronic-structural properties of the P-cluster and its function in electron transfer.^10^

Resolving the relationship between the magnetism and structure of the P-cluster, most notably for the metastable P^+^ state, is important for elucidating a complete mechanistic understanding of the P-cluster in the nitrogenase electron transfer cycle. Herein, we address this goal by combining light-controlled reduction of MoFe protein in complexes with cadmium sulfide nanocrystals (CdS)^16,17^ with EPR to resolve magnetic changes in the P-cluster during electron transfer that arise from discrete electronic-structural intermediates in the reduction of P^2+^ to P^+^.

An oxidized sample of nitrogenase MoFe protein was mixed with mercaptopropionic acid capped CdS quantum dots (Figure S1; see Supporting Information for details), and the P-cluster P^2+^ oxidation state was verified by EPR (Figure S2). The CdS:MoFe protein complexes were illuminated with a 405 nm LED at either 231 K or 263 K and then allowed to anneal in the dark at 236 K or 263 K, respectively, to prevent further light-driven reduction. By illuminating at subambient temperatures, the light-driven redox (i.e., electron transfer) process is decoupled from temperature sensitive chemical (i.e., ligand switching) steps during electron transfer and P-cluster conversion from P^2+^↔P^+^. As shown in Figure 2A, illumination at 263 K for 12 min resulted in reduction of the P-cluster exemplified by loss of P^2+^ intensity (36%, Figure S2 and Table S1). This change coincided with the appearance of an *S* = 1/2 rhombic signal at *g* = 2.05, 1.94, 1.81 (P^+^_1.81_, Figure 2A)^13,15,18^ and an *S* = 1/2 axial signal with *g* = 2.006, 1.89 (assigned to P^+^_1.89_, Figure 2B).^13,14^ Dark annealing at 263 K for 20 min (Figure 2A, green trace) led to complete loss of the P^+^_1.89_ signal, and an increase in amplitude of the P^+^_1.81_ (21%) and P^2+^ (7%) signals (Table S1).

**Figure 2:**
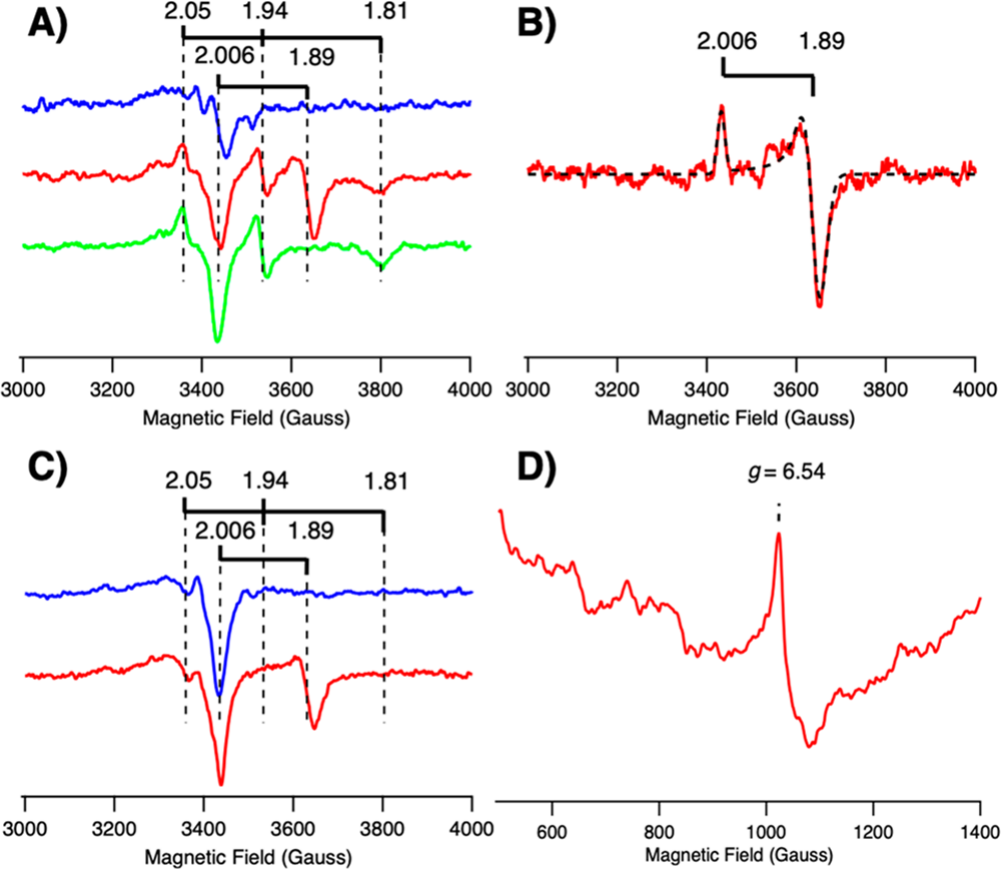
Illumination and EPR spectra of CdS:MoFe protein complexes at 263 K and 231 K. (A) *T* = 263 K. Blue trace, oxidized CdS:MoFe protein complexes. Red trace, after 12 min illumination at 263 K. Green trace, spectrum after incubation in the dark at 263 K for 20 min. (B) Illuminated (red trace) minus dark (green trace) difference spectrum. Simulation (black dashed trace) using *g* = 2.006, 1.89 assigned to the *S* = 1/2, P^+^_1.89_ signal. Buffer pH = 7; EPR conditions, *T* = 12 K, microwave power = 1 mW. (C) *T* = 231 K. Blue trace, oxidized CdS:MoFe protein complexes. Red trace, after 15.5 min illumination at 231 K. (D) Low-field EPR spectrum showing the high-spin, *S* = 7/2, *g* = 6.54 signal assigned as P^+^_6.54_. Sample pH = 7. EPR conditions: (C) *T* = 12 K, microwave power = 1 mW, (D) *T* = 18 K, microwave power = 25 mW. Populations of EPR signals are summarized in Table S1.

When illuminated at a lower temperature of 231 K for 15.5 min, reduction of the MoFe protein P-cluster led to a decrease in the P^2+^ signal intensity (19%) and appearance of the P^+^_1.89_ signal, whereas formation of the P^+^_1.81_ signal was suppressed. Rather, a low-field inflection at *g* = 6.54 (Figure 2D) appeared, resembling other high-spin signals observed for MoFe protein (Table S2, Figure S3). Rhombogram analysis and simulation of the *g* = 6.54 signal (referred to as P^+^_6.54_) indicates that it originates from a *S* = 7/2 spin system with E/D ≈ 0.024 (D = −3.2 cm^–1^) of the reduced P-cluster, where E and D^19^ are the zero-field splitting parameters (see Figure S3 for details).

Dark annealing at 236 K of the CdS:MoFe protein complexes illuminated at 231 K (Figure 2C) was used to monitor relative intensities of P^+^ intermediates following light-driven electron transfer to MoFe protein (Figure S4). EPR spectra of CdS or MoFe protein alone, before and after illumination and annealing, or of CdS:MoFe protein prior to illumination, did not produce any detectable signal changes (Figure S5). Simulations of the low-field regions using singular value decomposition (SVD, Figure S6) and the high-field region using EasySpin^20^ (Table S3, Figure S7) enabled time-dependent changes in signal intensities of P^+^ intermediates (P^+^_1.89_, and P^+^_1.81_ and P^+^_6.54_) to be fit to reaction models (Tables S4 and S5, Figure S8). The P^+^ signal intensity versus annealing time best fit to a three-step reaction model as summarized in eqs 2 and 3:
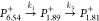
2
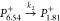
3

The fit shown in Figure 3 gave relative values for rate constants where *k*_2_ > *k*_1_ > *k*_3_ and predicts the high-spin P^+^_6.54_ P-cluster intermediate originates together with P^+^_1.89_ under photochemical reduction of the P^2+^ state (Figure 4). In the dark, P^+^_6.54_ partitions rapidly between P^+^_1.89_ or P^+^_1.81_. Therefore, a lack of P^+^_6.54_ under illumination at 263 K (Figure 2) is likely due to more rapid conversion to either P^+^_1.81_ or P^+^_1.89_ (*k*_1_ and *k*_2_ > *k*_3_) than at 231 K.

**Figure 3:**
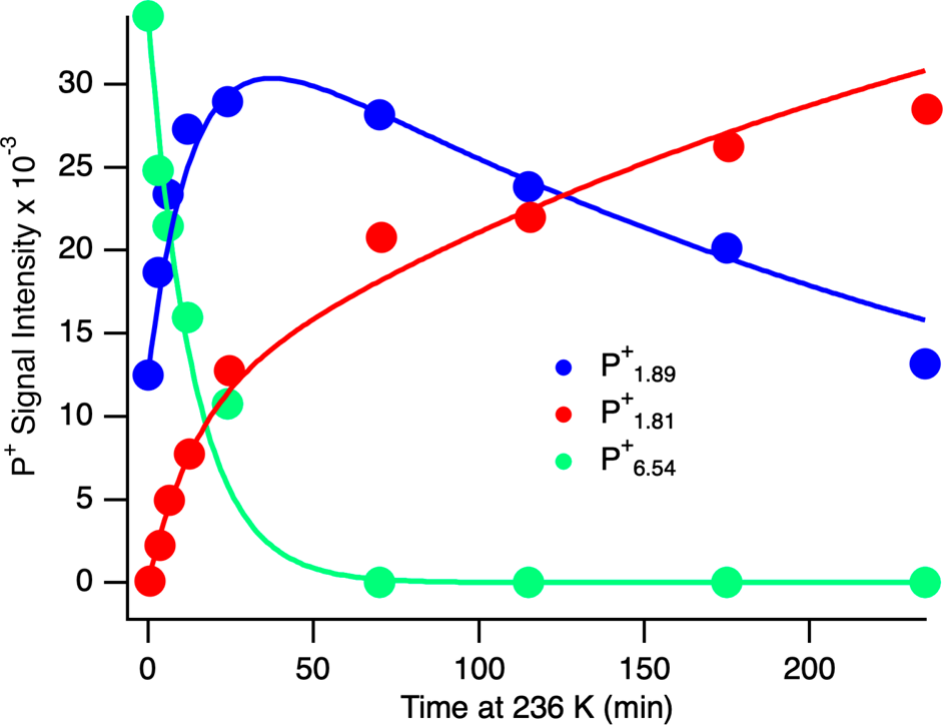
Time-dependent changes of the P^+^ EPR signals intensities in CdS:MoFe protein complexes. Initial (time = 0 min) P^+^ signal intensities were collected at 231 K. Changes are plotted versus time under dark annealing at 236 K. P^+^ signal intensities were determined using EasySpin and SVD analysis (see Supporting Information, Figures S6 and S7).^20^ Solid lines are fits of the experimental data to differential equations; dP^+^_1.89_/d*t* = *k*_*1*_[P^+^_6.54_] – *k*_*3*_[P^+^_1.89_], dP^+^_1.81_/d*t* = *k*_*2*_[P^+^_6.54_] + *k*_*3*_[P^+^_1.89_], and dP^+^_6.54_/d*t* = −(*k*_*1*_ + *k*_*2*_)[P^+^_6.54_] (Table S4). Green, P^+^_6.54_; blue, P^+^_1.89_; red, P^+^_1.81_.

**Figure 4:**
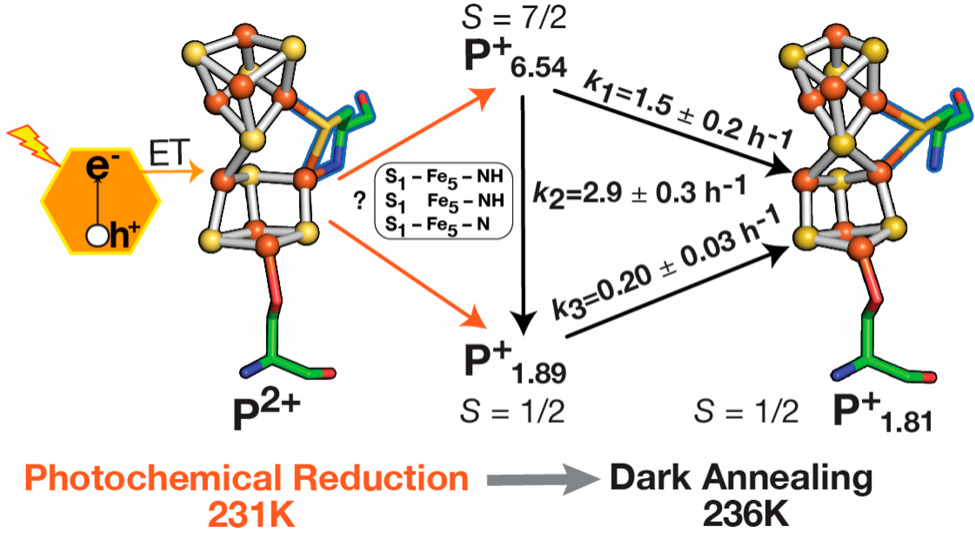
Schematic representation of the P^2+^ to P^+^ conversion in low temperature photochemical reduction of the MoFe protein P-cluster. Photoexcitation at 231 K of CdS:MoFe protein complexes poised in P^2+^ (left) leads to electron injection into the P-cluster and reduction to a mixed population of P^+^ states; the *S* = 7/2 P^+^_6.54_ and *S* = 1/2 P^+^_1.89_, which are based on the reaction model (Figure 3), correspond to distinct conformers (inset). Dark annealing at 236 K results in the conversion of P^+^_6.54_ to either P^+^_1.89_ (faster) or P^+^_1.81_ (slower), and conversion of P^+^_1.89_ to P^+^_1.81_. The rate constants of the conversion of P^+^ states are obtained from fits shown in Figure 3 to a reaction model in Table S4.

Dark annealing was performed over a range of 231 K to 245 K to obtain the temperature-dependence of *k*_3_ for the P^+^_1.89_↔P^+^_1.81_ step (eq 3, Figure S9, Table S6). An Arrhenius plot of ln *k*_3_ vs 1/*T* gave a value of *E*_a_ = 24 ± 8.3 kcal mol^–1^. The value suggests the P^+^_1.89_↔P^+^_1.81_ involves structural changes in MoFe protein at the P-cluster. For example, reductive formation of P^+^_1.81_ from P^2+^ at 298 K is pH-dependent (Figure 1) and is favored at pH ≈ 6 and nearly undetectable at basic pH (>8).^15^ Likewise, chemical oxidation of MoFe protein P-cluster from P^N^↔P^+^ at 298 K led to formation of both P^+^_1.81_ and P^+^_1.89_,^13^ with P^+^_1.89_ intensity being maximal at pH 8.4.^14^ The two results are consistent with formation of P^+^_1.89_ being reversible and both pH- and temperature-dependent.

In addition to analysis of the P^+^_1.89_ intermediate, photochemical reduction of MoFe protein at 231 K also enabled assignment of the P^+^_6.54_ high-spin state to a unique electron transfer intermediate (Figure 3). In the P^2+^↔P^+^ reduction step, the high-spin P^+^_6.54_ state has two possible fates: direct conversion to P^+^_1.81_ (eq 3) where *k*_2_ > *k*_3_ or rapid conversion to P^+^_1.89_ followed by slow P^+^_1.89_↔P^+^_1.81_ conversion (eq 2). Thus, the reaction model for the P-cluster P^2+^↔P^+^ conversion, summarized in Figure 4, involves two spin-state isomer intermediates. The observation of multiple electronic intermediates associated with a redox step in the P-cluster is similar to the observation of low-spin and high-spin S_2_ states of the PSII oxygen evolving complex that arise from valence isomerism in Mn–O–Mn coordination from different S_2_ conformers that function in the catalytic cycle of water oxidation.^21−25^ The interconversion of P^+^_6.54_↔P^+^_1.89_ may likewise arise from conformational isomerism in Fe-coordination to S_1_ (see Figure 1) that guide formation of P^+^ with surrounding structural changes. Overall, the results from combining low temperature photochemical reduction of the MoFe protein with dark annealing reveal that formation of the metastable P^+^ state, P^+^_1.81_, involves intermediate spin states and electronic configurations that occur with changes in P-cluster coordination.

As established by kinetic and theoretical studies, correlated motions within the nitrogenase complex during turnover have an important function in enabling P-cluster mediated electron transfer to be integrated with catalysis.^7−9,26^ As shown here, during electron transfer, there are also discrete changes in P-cluster magnetic structure that are linked to changes in oxidation state. The EPR analysis and kinetic model are most consistent with these magnetic states originating from different P-cluster conformers during electron transfer and reduction of P^2+^ to P^+^, which may function in the electron transfer mechanism within the nitrogenase complex during ammonia production.^27^

## Supporting Information Available

A description of experimental procedures including nanocrystal synthesis, EPR spectroscopy and simulation, and reaction models. Figures of parallel mode and low-field perpendicular mode EPR, a detailed analysis for the high-field P^+^_6.54_ signal assignment, and temperature-dependence analysis of P^+^_1.89_↔P^+^_1.81_ EPR signals; Tables of spectral analysis and reaction model fits (PDF).
