# Supplementary material for: Dissecting Electronic-Structural Transitions in the Nitrogenase MoFe Protein P-Cluster during Reduction
Source: J Am Chem Soc. 2022 Mar 22;144(13):5708–12. doi: 10.1021/jacs.1c13311 (PMC8991001; doi:10.1021/jacs.1c13311)
Supplement: Supplementary file 1 — ja1c13311_si_001.pdf [file ja1c13311_si_001.pdf]

Supporting Information

Dissecting Electronic-Structural Transitions in the Nitrogenase MoFe Protein P-cluster During Reduction

Bryant Chica[a], Jesse Ruzicka[b], Lauren M. Pellows[b], Hayden Kallas[c], Effie Kisgeropoulos[a], Gregory E. Vansuch[a], David W. Mulder[a], Katherine A. Brown[a], Drazenka Svedruzic[a], John W. Peters[d], Gordana Dukovic[b,e,f], Lance C. Seefeldt[c], Paul W. King[a,e]*

[a]Biosciences Center, National Renewable Energy Laboratory, Golden, Colorado 80401, United States.

[b]Department of Chemistry, University of Colorado Boulder, Boulder, Colorado 80309, United States.

[c]Department of Chemistry and Biochemistry, Utah State University, Logan, Utah 84322, United States.

[d]Institute of Biological Chemistry, Washington State University, Pullman, Washington 99163, United States.

[e]Renewable and Sustainable Energy Institute (RASEI), University of Colorado Boulder, Boulder, Colorado 80309, United States.

[f] Materials Science and Engineering, University of Colorado Boulder, Boulder, Colorado 80303, United States.

Materials and Methods

Nanocrystal Synthesis

The CdS quantum dots (QDs) were synthesized and purified using a procedure adapted from Pearce et al.[1] The QD synthesis and purification procedure follows the previously reported in Chica et al.[2] After the sulfur precursor injection, the QDs were allowed to grow for 45 s before cooling the reaction flask in a mineral oil bath to arrest the reaction. The QD diameter was determined using the position of the first exciton peak (412 nm) and sizing curves found in Yu et al.,[3] giving a CdS QD diameter of 3.7 nm (Figure S1).

All experiments were performed using QDs capped with 3-mercaptopropionic acid (3-MPA, Strem Chemicals, ≥99%) ligands. The native oleate ligands were replaced with 3-MPA following a previously reported procedure.[1-3] The ligand exchanged QDs were redissolved in Millipore® water. The concentration of the QD solution was determined by using Lambert-Beer Law using an Agilent Cary 60 UV-Vis spectrophotometer equipped with a xenon source lamp. The UV-Vis sample was prepared in a 1 cm quartz cuvette in inert argon atmosphere glovebox. Molar absorptivity was determined from the QD diameter using the methods of Yu et al.[3]

MoFe Protein Preparation

MoFe protein was prepared by expression in *Azotobacter vinelandii* (A.v) and purified using nickel affinity chromatography as described previously.[4-5] Purified proteins were concentrated using a Millipore solvent-resistant stirred cell under an Ar atmosphere with appropriate molecular weight cutoff filters. MoFe protein purity, concentration and activity were assessed as previously described.[2]

Preparation of CdS:MoFe Protein Complexes

All sample manipulations were performed in a nitrogen containing anaerobic chamber (Mbraun). An aliquot of wild-type MoFe protein was exchanged into pH = 7 MOPS buffer (100 mM MOPS, 200 mM NaCl, 5% Glycerol) using centrifugal molecular weight cutoff filters (Amicon Ultra) to a concentration of 48 μM and 200 μl. The EPR spectrum was taken at 12K and 1mW power the sample was titrated with 3 mM Indigo disulfonic acid (IDS) with mixing. The titration was continued until the S=1/2, g = 1.81 rhombic signal of the P-cluster P^+ state was no longer observed, indicating oxidation of the P-cluster to P^{2+}. The presence of P^{2+} was confirmed by measuring the parallel mode EPR spectrum (T=10K, and microwave power=100 mW) for the presence of the P^{2+} signal at g = 11.8.[6] The sample was then transferred from the EPR tube and IDS removed via size exclusion chromatography and sample exchange into buffer containing 100 mM MOPS, pH=7, 200 mM NaCl, 5 mM mercaptopropionic acid, and 5% Glycerol. One equivalent of mercaptopropionic acid capped 3.72 nm CdS nanocrystals (48 μM) was added, and buffer added to a total volume to 200 μl. Samples were transferred to a quartz EPR tube and kept in the dark in liquid nitrogen.

Illumination and Dark Annealing of EPR Samples

A specialized device, pictured above, was constructed for illumination and dark annealing of CdS:MoFe protein samples for EPR analysis. Briefly, a computer controlled liquid nitrogen cryostat (ER 4131 VT, Bruker) was used for sample temperature control. The quartz sample holder portion of the cryostat was mounted inside an integrating sphere (4p4, ThorLabs) mounting hardware was constructed at an in-house machine shop. A 1W 405 nm LED source (M405L4 ,Thorlabs) was interfaced directly to the integrating sphere to facilitate unform excitation of the EPR samples under strict temperature control. Sample temperature was calibrated using a thermocouple mounted directly inside a buffer containing EPR tube to directly probe the conditions at the sample.

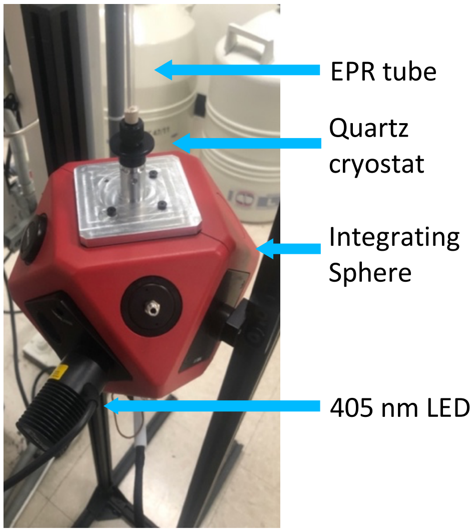

For illumination experiments, samples were loaded into the temperature calibrated cryostat, allowed to equilibrate for ~30 s then the 1W LED was turned on for the specified illumination period. After the specified period elapsed, the sample was rapidly withdrawn from the cryostat and immersed in liquid nitrogen within ~ 1-2 s. In the dark annealing experiments, samples were transferred from liquid nitrogen to the temperature calibrated cryostat for the specified dark annealing time period. During this time the LED source is in the off state and room lights were dimmed as much as possible to prevent any effect from ambient lighting. After the specified dark annealing time period, samples were rapidly withdrawn and quenched in liquid nitrogen.

EPR Spectroscopy

EPR spectroscopy was carried out as previously decribed.[2] EPR spectra were collected on a Bruker E-500 EPR spectrometer equipped with an in-cavity cryogen-free cryostat, and Mercury iTC temperature controller (Oxford Instruments). Modulation frequency was set at 100 kHz with a modulation amplitude of 10 Gauss. Baseline and cavity corrections were applied using Igor Pro version 8. EPR spectra shown in Figures 2, S2, S5 and S7 were collected using a dual parallel/perpendicular mode resonator (Bruker model DM4112). The remaining EPR spectra were collected in perpendicular mode using a Bruker model SHQ resonator.

Determination of Time-Dependent Changes of P-cluster $P^{+}_{6.54}$ Signal Intensity Under Dark Annealing

Changes in the low-field region were extracted by singular value decomposition of the dark annealing EPR spectra in the low-field region, between 500 Gauss and 1200 Gauss, using timepoint spectra collected at 25 mW and T=12 K (Figure S4). Analysis was performed using a script written in the programming language, R (available upon request). The vectorial component that reproduced the $P^{+}_{6.54}$ signal (Figure S6) was selected and the time-dependent amplitude vector (i.e., temporal profile) corresponding to that singular vector was used to provide the change in the $P^{+}_{6.54}$ signal intensity for each timepoint spectrum. Beyond 70 min the $P^{+}_{6.54}$ signal has completely decayed and becomes undetectable. The points between 100 min and 235 min

were averaged and that value was used as a vertical offset to baseline the points between 100 min and 235 min at zero. This baseline treatment did not alter resulting rate constants from fitting the differential equation of the reaction model in Table S4.

Determination of Temperature Dependence of the $\mathrm{P}^{+}_{1.89} \overset{k_3}{\rightarrow} P^{+}_{1.81}$ Reaction

To determine the energy of activation for the $\mathrm{P}^{+}_{1.89} \overset{k_3}{\rightarrow} P^{+}_{1.81}$ reaction, the change in $P^{+}_{1.89}$ signal intensity was measured in CdS:MoFe protein complexes prepared under illumination at 231K under dark annealing at 236K, 240K and 245K by measuring the peak to trough amplitude of the *g* = 1.89 inflection. The P-cluster in the CdS:MoFe protein was poised in $P^{+}_{1.89}$ and $P^{+}_{1.81}$ to selectively measure k_3 in the absence of contributions to changes in $P^{+}_{1.89}$ and $P^{+}_{1.81}$ by $P^{+}_{6.54}$. The time-dependent decrease in $P^{+}_{1.89}$ signal intensities under dark annealing at 236 K, 240 K and 245 K were fit to the equation "$P^{+}_{1.89} = k_3 * t$". Values obtained for k_3 are in Table S6.

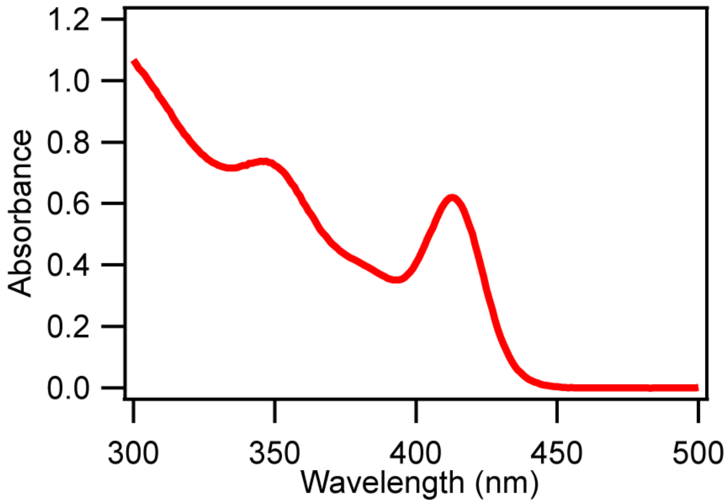

Figure S1: UV-Vis Absorption Spectrum of CdS Quantum Dots.

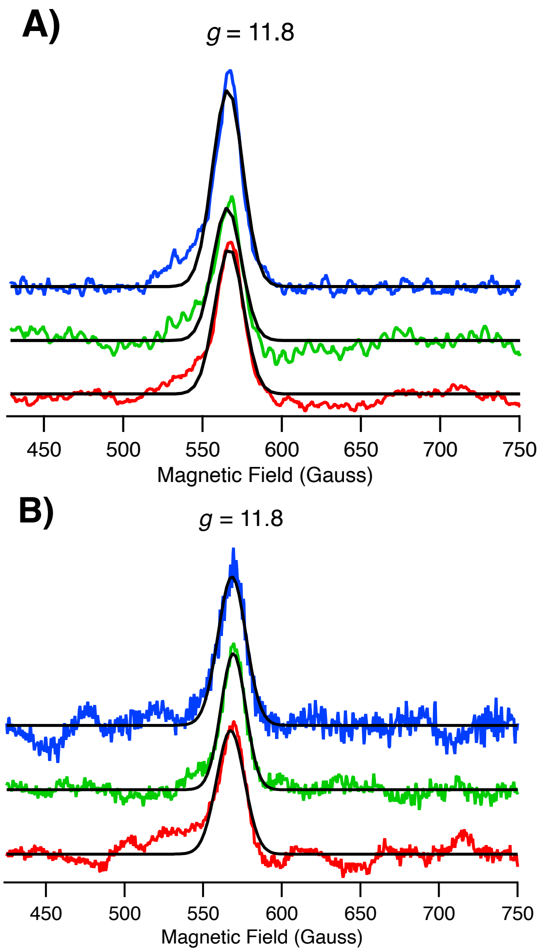

Figure S2. Changes in the g = 11.8 P^{2+} parallel mode EPR signal upon illumination of CdS:MoFe protein complexes and dark annealing. **A**) Blue trace, initial spectrum of oxidized CdS:MoFe protein complexes. Middle, green trace EPR spectrum after 720 s of illumination at 263 K. Red trace, spectra after dark annealing for 20 min at 263 K. Spectra were baseline corrected using a spline function with the baseline fit excluding the region 500 - 650 Gauss. **B**) Blue trace, initial spectrum of oxidized CdS:MoFe protein complexes. Green trace, spectrum after 930 s of illumination at 231K. Red trace, spectrum of sample after dark annealing for 4 h at 236 K. Black traces in **A** and **B** are fits to gaussian profiles in Igor Pro. EPR conditions; Parallel mode, T=10 K, microwave power = 100 mW.

Table S1. Effect of temperature and illumination on P-cluster EPR signals in CdS:MoFe protein complexes.

**Reaction conditions**[a]	**Changes in P-cluster spin state populations**[b,c]			
	**P^{2+} (*S*=4) *g* = 11.8**	**P^{+} (*S*=1/2)** [d]		**P^{+} (*S*=7/2)**
		***g* = 1.81**	***g* = 1.89**	***g* = 6.54**
Pre-illumination @ 263 K	100 ± 3%	0%	0%	0%
Illumination @ 263 K, 12 min	64 ± 6%	79%	100%	0%
Dark anneal @263 K, 20 min	73 ± 5%	100%	0	0%
Pre-illumination @ 231 K	100 ± 15%	0%	0%	0%
Illumination @ 231 K, 15.5 min	81 ± 9%	0	43%	100%
Dark anneal @236 K, 235 min	82 ± 16%	100%	21%	0%

[a] Illumination by 1 W 405 nm LED. Illumination at 263 K was for carried out for 12 min, and illumination at 231 K was carried out for 15.5 min. Annealing was performed in the dark at the specified temperature.

[b] Values are relative to maximal signal intensity.

[c] Uncertainties computed from propagated error (standard deviation) in the area (amplitude x width) of the gaussian fits.

[D] P^{+} populations derived from simulated EPR spectra using EasySpin, errors in individual species are not simulated.

Table S2. High-spin states of the P-cluster P^+ state observed in MoFe protein.[a]

MoFe protein[a] and pH conditions	EPR signal *g* values[b]	Assigned spin states	E/D values	Refs
β–S188C[c] pH = 7	7.7, 6.7, 5.3	5/2	N.R.	Chan and Seefeldt, 1999[7]
Wild-type pH = 7.4	7.3<br>6.67, 5.3	5/2<br>5/2	0.059<br>0.029	Tittsworth and Hales 1993[6]
Wild-type pH = 8.34	7.3, 6.54, 5.24	N.D.	N.D.	this work
CdS:MoFe protein complexes pH = 7	9.8, 6.54	7/2	0.024	this work

[a] MoFe protein from *Azotobacter vinelandii*.
[b] *g* values assigned to the P-cluster spin states.
[c] β–S188C is the MoFe protein with a serine to cysteine mutation at position 188 of the β-subunit.[7]
N.R., Not Reported.
N.D., Not Determined.

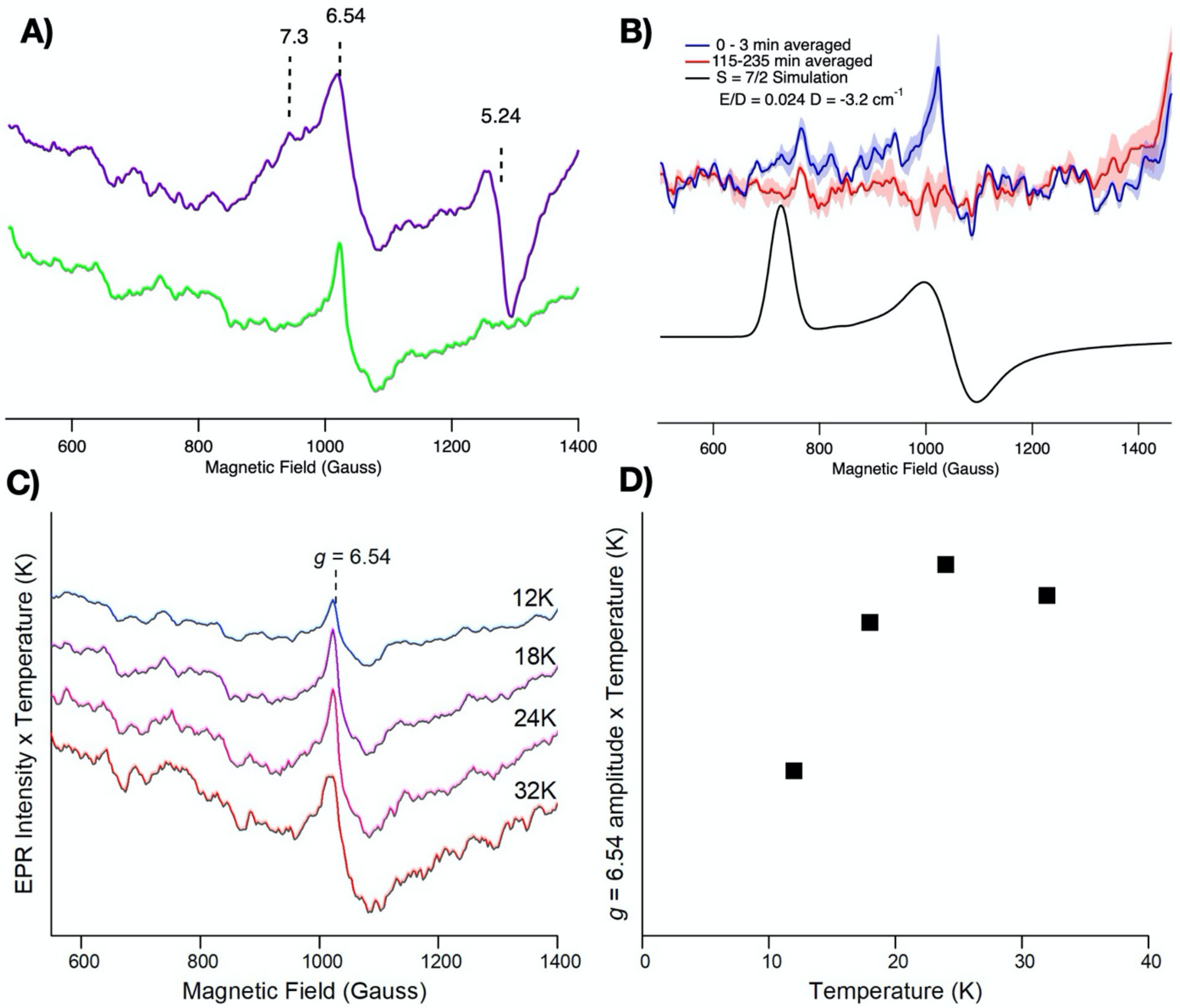

Figure S3. Low-field EPR spectra used for the assignment of the $P^{+}_{6.54}$ signal.

A) Purple trace. Low-field X-band EPR spectrum of MoFe protein alone and poised in the P^+ state. MoFe protein in the P^+ state has been shown to produce low-field inflections, the intensity of which vary with pH, and are summarized in Table S2. Spin state assignment in this region is complicated by low intensities due to broad spectral width and distorted line shapes that arise from wide distribution in the zero field splitting due to differences in cluster geometry and represented by differences in the measured E and D values.[8] In the case of the MoFe protein this is further complicated by the observation that the low-field spectrum of P^+ can give rise to at least 2 high-spin states that are in equilibrium as is shown here.[6-7] The overlapping signals at X-band frequency consist of the three components of $g \approx 7.3$, 6.5 and 5.2, and were assigned as two S = 5/2 states.[6] **Green trace**. The low-field EPR spectrum of the CdS:MoFe protein complexes poised in P^{2+} and illuminated for 15.5 min with 405 nm light at T=231 K, showing only a g = 6.54 signal and no g = 7.3 or 5.24 signal.

B) Blue trace. An average signal for the 0-3 min annealing period, the g = 6.54 is observed to be maximal intensity, and to coincide with a g = 9.8 signal (shading +/- 1 standard deviation)**. Red trace.** The region of the g = 6.54 and 9.8 signals averaged for the final 115-235 min period of dark annealing. Based on deconvolution of g = 6.54 from g = 7.3 and g = 5.25 (**panel A, green trace**), and being coincident with g = 9.8, a simulation of g = 9.8–6.54 was performed in EasySpin for a S = 7/2 spin system. When defining the

spin system in the simulation, rhombogram analysis, coincident with analysis of the temperature dependance of the g = 6.54 signal as described below, was used to assign the signal to the excited |± ½ > doublet state of a S = 7/2 spin system. The simulation produced an E/D ≈ 0.024 (D = -3.2 cm^{-1}) and showed significant D-strain broadening and distortion in the spectral lineshape.[8]

C) Temperature dependance of the low-field spectra of oxidized CdS:MoFe protein complexes after 15.5 min of 405 nm illumination at 231 K (spectra recorded at 25 mW microwave power).

D) Temperature dependance of the g = 6.54 signal amplitude as determined by the peak-to-peak height and correcting for the Curie law by multiplying by the absolute temperature. The increased signal amplitude in the 12-24 K range indicates the low-field signal arises from an excited state with D < 0. Comparison of spectra recorded at 12 K and either 1 or 25 mW microwave power show no power-saturation further indicating the exited state nature of the species. These observations are consistent with simulation in (**B**) that reproduced the spectral feature at g = 6.54 from the |± ½ > doublet of a S = 7/2 spin system with E/D = 0.024 (D = -3.2 cm^{-1}).

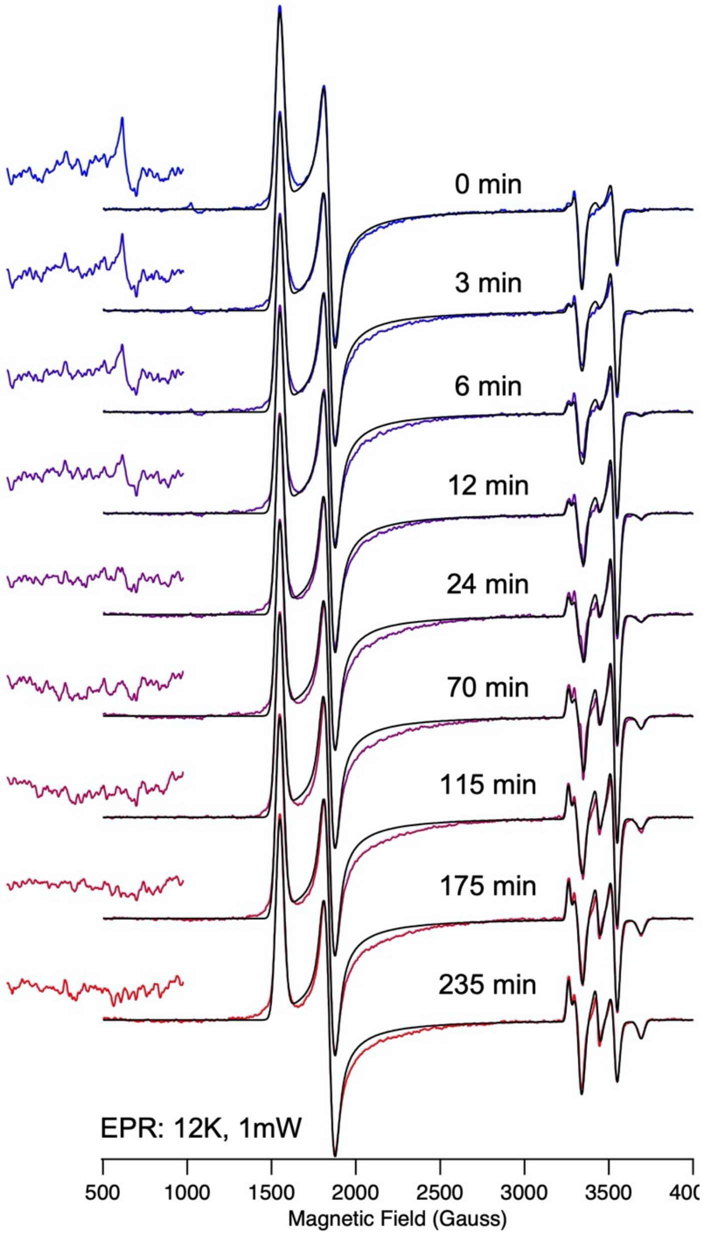

Figure S4. EPR spectra of CdS:MoFe protein complexes, illuminated at 231 K for 15.5 min (spectrum is shown at the top as “0 min”), and subjected to dark annealing at 236K for specified time periods from 0 to 235 min (blue to red traces). Fits generated in EasySpin are shown as black traces[9] obtained by modelling the spectra using the E_0, $P^{+}_{1.89}$ and $P^{+}_{1.81}$ and P^{3+} signal components in Table S3. The low-field region containing $P^{+}_{6.54}$ is shown magnified for each timepoint and illustrates data used for the SVD analysis. EPR conditions, T=12 K, microwave power, 1 mW.

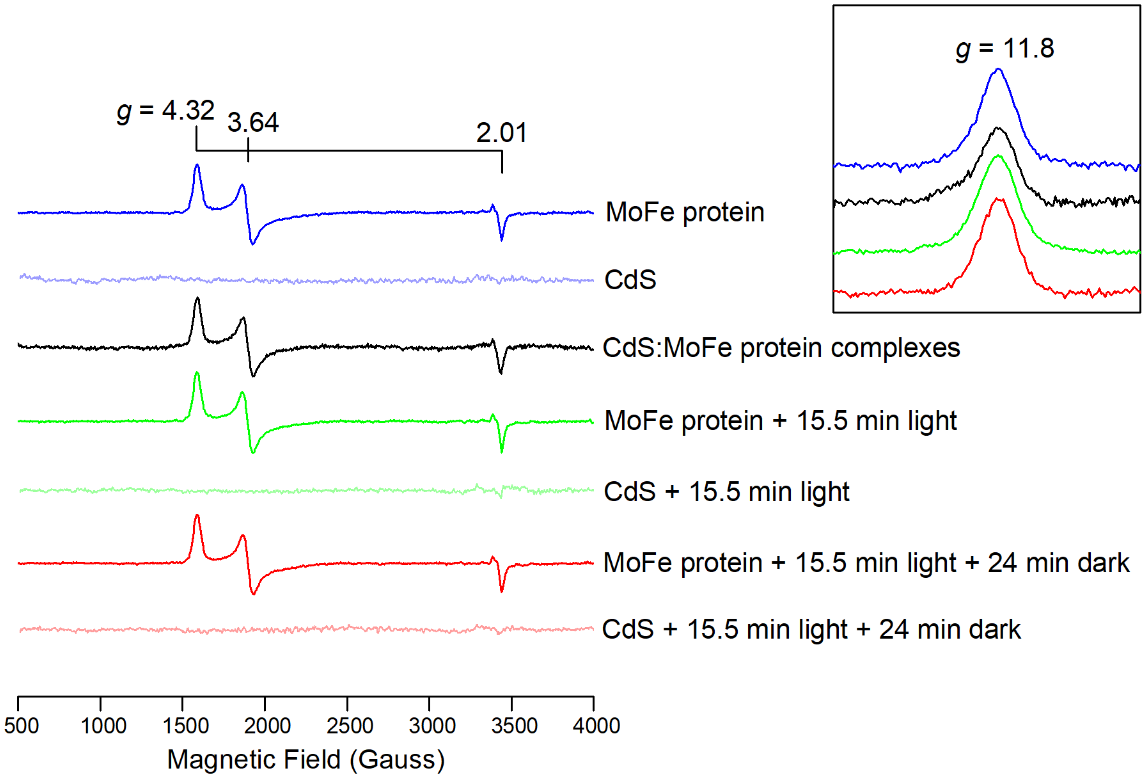

Figure S5. EPR spectra of oxidized MoFe protein (100 μM, blue trace), CdS quantum dots (48 μM, blue trace, light), and CdS:MoFe protein complexes (100 μM each, black trace) without illumination. MoFe protein alone or CdS nanoparticles alone were subjected to identical illumination (405 nm for 15.5 min at 231 K) and annealing conditions (dark for 24 min at 236 K) as CdS:MoFe protein complexes (*see* Figure S4). No significant spectral changes were observed compared to the un-illuminated samples. Spectra are comprised of an S = 3/2 FeMo-co signal (measured under perpendicular mode) reflective of the E_0 state (g = 4.32, 3.64, 2.01) and a signal at g = 11.8 (inset, measured under parallel mode) reflective of the P-cluster poised in the P^{2+} state. EPR conditions, T = 12 K, microwave power = 1 mW (100 mW, inset), signal intensities scaled for protein concentration, spectra baseline and cavity corrected. MoFe protein alone and CdS quantum dots alone prepared in 100 mM MOPS, 200 mM NaCl, 5 mM MPA, 5% glycerol.

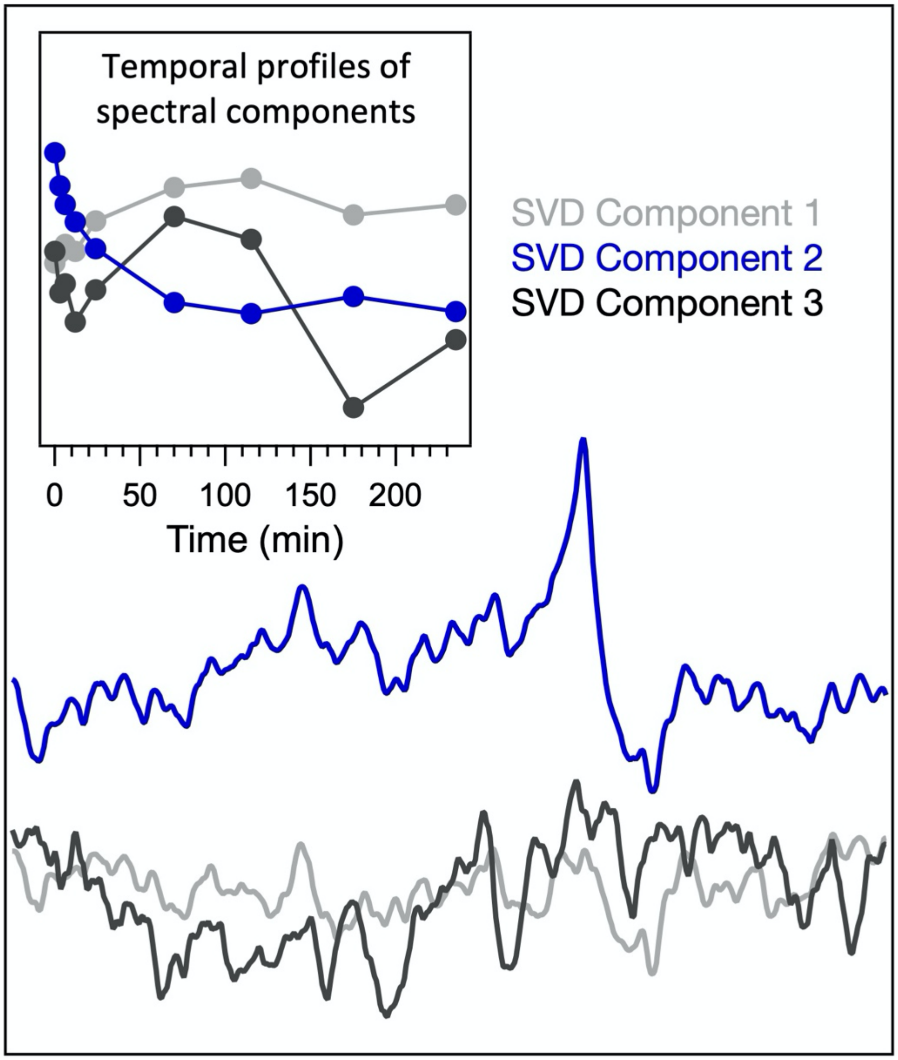

Figure S6. Singular value decomposition (SVD) analysis. The output of SVD analysis includes three matrices (*U, S, V*). The *U* matrix consists of the individual spectral components extracted from the experimental data (*main figure; solid lines*), while the *V* matrix holds the corresponding temporal profiles of each spectral component (*inset; lines with markers*); the *S* matrix provides the singular values or "weights" associated with each component.[10]

SVD analysis performed on the low-field region produced only one component of quality (*blue line*), reproducing nicely the $P^{+}_{6.54}$ signal features. The other components (*gray and black lines*) represent the remaining noise in the spectrum. This is corroborated by examining the time-dependent profiles, as only the profile associated with the "$P^{+}_{6.54}$ – like" spectral component (*inset, blue circles*) exhibits a clear decay from 0 to 70 min that matches to the change in the g = 9.8-6.54 region shown in Figure S4. Although the weights of components 1 and 2 were similar in this analysis, additional analyses (e.g., using other data sets, two instead of three components) reproduced the single component of quality observed here with analogous amplitude vectors.

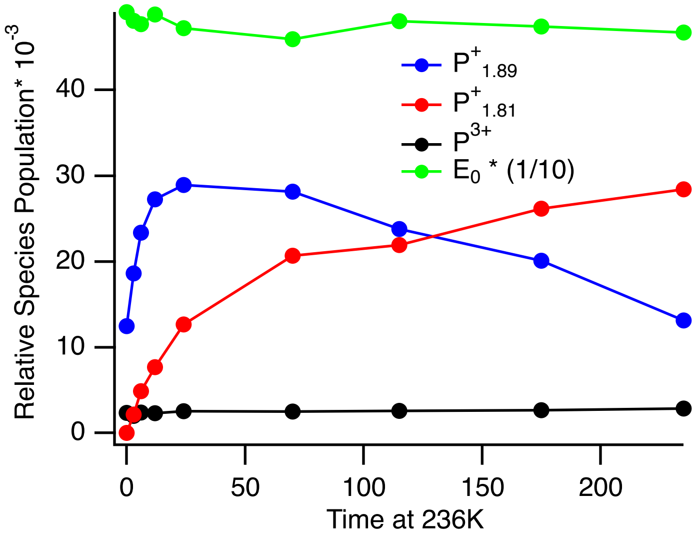

Figure S7. Time-dependent changes of the P^+, P^{3+} and E_0 populations obtained by simulation of the spectra in Figure S4 during dark annealing at 236 K. Simulations were performed in EasySpin using the MoFe protein E_0, The $P^+_{1.89}$ and $P^+_{1.81}$ and P^{3+} signals are identified in Table S3.[9]

Table S3. Signals used to model EPR spectra in Figure S4.

MoFe protein cluster, EPR signal	*g*-values
E_0, rhombic[a]	4.32, 3.64, 2.01
P^{3+}, axial	2.03, 2
$P^+_{1.81}$, rhombic	2.05, 1.95, 1.81
$P^+_{1.89}$, axial	2.006, 1.89

[a] S = 3/2 state modeled as a pseudo, S = ½ signal with specified *g*-values.

Table S4. Fitting parameters for a two-sequence reaction model for the EPR changes in photochemically reduced CdS:MoFe protein complexes under dark annealing at 236 K.

Reaction step[a]	Differential equation[b]	Rate constant (h^{-1})
$P^+_{6.54} \rightarrow P^+_{1.89}$<br>$P^+_{6.54} \rightarrow P^+_{1.81}$	$dP^+_{6.54}/dt = -(k_1 + k_2)[P^+_{6.54}]$	$k_1 = 2.9 \pm 0.3$<br>$k_2 = 1.5 \pm 0.2$
$P^+_{6.54} \rightarrow P^+_{1.89}$<br>$P^+_{1.89} \rightarrow P^+_{1.81}$	$dP^+_{1.89}/dt = k_1[P^+_{6.54}] - k_3[P^+_{1.89}]$	$k_1 = 2.9 \pm 0.3$<br>$k_3 = 0.2 \pm 0.02$
$P^+_{6.54} \rightarrow P^+_{1.81}$<br>$P^+_{1.89} \rightarrow P^+_{1.81}$	$dP^+_{1.81}/dt = k_2[P^+_{6.54}] + k_3[P^+_{1.89}]$	$k_2 = 1.5 \pm 0.2$<br>$k_3 = 0.2 \pm 0.02$

[a]Reaction Model: $P^+_{6.54} \overset{k_1}{\rightarrow} P^+_{1.89} \overset{k_3}{\rightarrow} P^+_{1.81}$ and $P^+_{6.54} \overset{k_2}{\rightarrow} P^+_{1.81}$

[b]The fit was performed using the R package deSolve as detailed previously.[2] As shown in the spectral fits in Figure S7, the intensity of P^{3+} and E_0 signals did not vary under dark annealing and were not included in the model.

Table S5. Fitting parameters for a single sequential reaction model to the EPR changes in photochemically reduced CdS:MoFe protein under dark annealing.

**Reaction step**[a]	**Differential equation**[b]	**Rate constant (h^{-1})**
$P^+_{6.54} \rightarrow P^+_{1.89}$	$dP^+_{6.54}/dt = -k_1[P^+_{6.54}]$<br>$dP^+_{1.89}/dt = k_1[P^+_{6.54}] - k_2[P^+_{1.89}]$	$k_1 = 3.5 \pm 0.5$<br>$k_2 = 0.4 \pm 0.03$
$P^+_{1.89} \rightarrow P^+_{1.81}$	$dP^+_{1.81}/dt = k_2[P^+_{1.89}]$	$k_2 = 0.4 \pm 0.03$

[a] Reaction model: $P^+_{6.54} \xrightarrow{k_1} P^+_{1.89} \xrightarrow{k_2} P^+_{1.81}$

[b] The fit was performed using the R package deSolve as detailed previously.[2] As shown in the spectral fits in Figure S7, the intensity of P^{3+} and E_0 signals did not vary under dark annealing and were not included in the model.

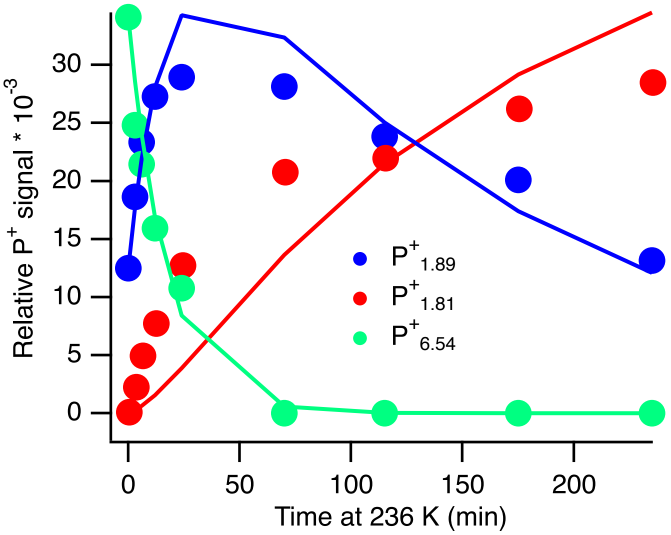

Figure S8. Time-dependent changes of the P^+ signal populations in MoFe protein complexes illuminated at 231 K and analyzed under dark annealing at 236 K. The non-interpolated fits of the experimental data to differential equations for a sequential reaction model (Table S5) are shown as solid lines. Green = $P^+_{6.54}$, blue = $P^+_{1.89}$, and red = $P^+_{1.81}$. Differential equation used for fits; $dP^+_{6.54}/dt = -k_1[P^+_{6.54}]$; $dP^+_{1.81}/dt = k_2[P^+_{1.89}]$; and $dP^+_{1.89}/dt = k_1[P^+_{6.54}] - k_2[P^+_{1.89}]$ (Table S5).

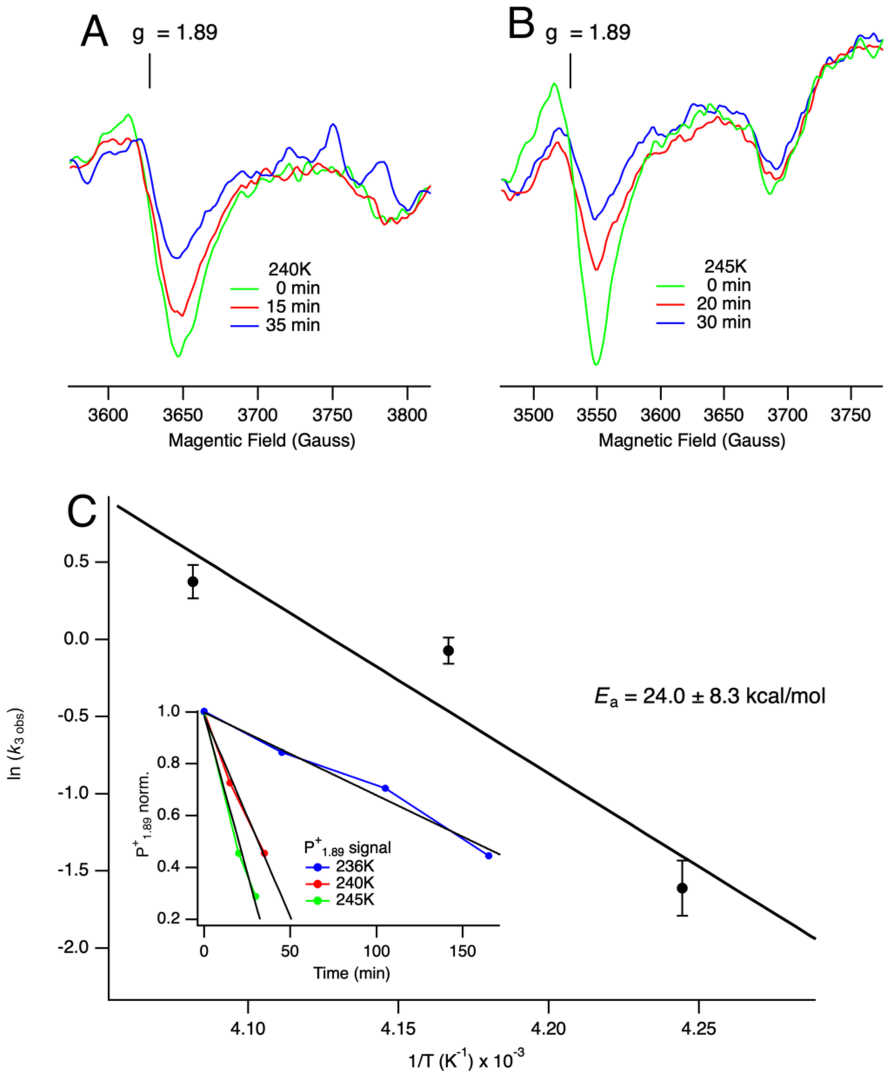

Figure S9. Time-dependent changes in the $P^+_{1.89}$ signal in MoFe protein complexes illuminated at 231 K and dark annealed at different temperatures. A) Change in $P^+_{1.89}$ intensity under dark annealing at 240 K. B) Change in $P^+_{1.89}$ intensity under dark annealing at 245 K. EPR spectra in panel A) were collected on a dual mode resonator, and spectra in panel B) were collected on perpendicular mode resonator (see methods for details). EPR conditions: T = 12 K , microwave power = 1 mW. C) Arrhenius plot of lnk_3 versus 1/T for $\mathrm{P}^+_{1.89} \xrightarrow{k_3} P^+_{1.81}$ reaction step. Black line is a linear fit where slope = E_a/R; (R = 0.001987 kcal * mol^{-1} *K) and error reported and one standard deviation. Inset: Time-dependent changes of the normalized $P^+_{1.89}$ signal during dark annealing at the indicated temperatures. Black overlay traces are linear fits with slope = -k_3. Values for k_3 are reported in Table S6, plus or minus one standard deviation. The change in $P^+_{1.89}$ signal intensity under dark annealing at 236 K is from the spectra shown in Figure S4.

Table S6. Temperature-dependent values of k_3 for $\mathrm{P}^+_{1.89} \xrightarrow{k_3} P^+_{1.81}$ determined from Figure S9.

**T (K)**	***K*₃ (h⁻¹)**
236	0.20 ± 0.04
240	0.93 ± 0.08
245	1.45 ± 0.15

References

1. Pearce, O. M.; Duncan, J. S.; Damrauer, N. H.; Dukovic, G., Ultrafast hole transfer from CdS quantum dots to a water oxidation catalyst. *J. Phys. Chem. C* **2018,** *122* (30), 17559-17565.

2. Chica, B.; Ruzicka, J.; Kallas, H.; Mulder, D. W.; Brown, K. A.; Peters, J. W.; Seefeldt, L. C.; Dukovic, G.; King, P. W., Defining Intermediates of Nitrogenase MoFe Protein during N2 Reduction under Photochemical Electron Delivery from CdS Quantum Dots. *J. Am. Chem. Soc.* **2020,** *142* (33), 14324-14330.

3. Yu, W. W.; Qu, L.; Guo, W.; Peng, X., Experimental determination of the extinction coefficient of CdTe, CdSe, and CdS nanocrystals. *Chem. Mats.* **2003,** *15* (14), 2854-2860.

4. Christiansen, J.; Goodwin, P. J.; Lanzilotta, W. N.; Seefeldt, L. C.; Dean, D. R., Catalytic and Biophysical Properties of a Nitrogenase Apo-MoFe Protein Produced by a nifB-Deletion Mutant of Azotobacter vinelandii. *Biochemistry* **1998,** *37* (36), 12611-12623.

5. Jiménez-Vicente, E.; Martin Del Campo, J. S.; Yang, Z.-Y.; Cash, V. L.; Dean, D. R.; Seefeldt, L. C., Chapter Nine - Application of affinity purification methods for analysis of the nitrogenase system from Azotobacter vinelandii. In *Methods Enzymol.*, Armstrong, F., Ed. Academic Press: 2018; Vol. 613, pp 231-255.

6. Tittsworth, R. C.; Hales, B. J., Detection of EPR signals assigned to the 1-equiv-oxidized P-clusters of the nitrogenase MoFe-protein from Azotobacter vinelandii. *J. Am. Chem. Soc.* **1993,** *115* (21), 9763-9767.

7. Chan, J. M.; Christiansen, J.; Dean, D. R.; Seefeldt, L. C., Spectroscopic Evidence for Changes in the Redox State of the Nitrogenase P-Cluster during Turnover. *Biochemistry* **1999,** *38* (18), 5779-5785.

8. Hagen, W. R., Wide zero field interaction distributions in the high-spin EPR of metalloproteins. *Mol. Phys.* **2007,** *105* (15-16), 2031-2039.

9. Stoll, S.; Schweiger, A., EasySpin, a comprehensive software package for spectral simulation and analysis in EPR. *J. Magn. Reson.* **2006,** *178* (1), 42-55.

10. Hendler, R. W.; Shrager, R. I., Deconvolutions based on singular value decomposition and the pseudoinverse: a guide for beginners. *J. Biochem. Biophys. Methods.* **1994,** *28* (1), 1-33.
